# A phase I study of liposomal Irinotecan (ONIVYDE®) in combination with TAS-102 (LONSURF®) in refractory solid tumors

**DOI:** 10.1007/s10637-025-01547-2

**Published:** 2025-06-04

**Authors:** Nai-Jung Chiang, Li-Yuan Bai, I.-Wei Ho, Chih-Hung Hsu, Yi-Hsin Liang, Chang-Fang Chiu, Ching-Chan Lin, Kwang-Yu Chang, Shang-Hung Chen, Hui-Jen Tsai, Yu-Ping Lin, Li-Tzong Chen, Chia-Chi Lin

**Affiliations:** 1 Department of Oncology, Taipei Veterans General Hospital, Taipei, Taiwan; 2 School of Medicine, College of Medicine, National Yang Ming Chiao Tung University, Taipei, Taiwan; 3 National Institute of Cancer Research, National Health Research Institutes, Tainan, Taiwan; 4 Division of Hematology and Oncology, Department of Internal Medicine, China Medical University Hospital and China Medical University, Taichung, Taiwan; 5 Departments of Oncology, College of Medicine, National Taiwan University Hospital and Graduate Institute of Oncology, National Taiwan University, Zhongshan S. Rd, Taipei, 10002 Taiwan; 6 Department of Oncology, College of Medicine, National Cheng Kung University Hospital, National Cheng Kung University, Tainan, Taiwan; 7 PharmaEngine, Inc., Taipei, Taiwan; 8 Kaohsiung Medical University Hospital, and Center for Cancer Research, Kaohsiung Medical University, Kaohsiung, Taiwan

**Keywords:** Liposomal irinotecan, TAS-102, Solid tumors, Phase 1 clinical trial, Pharmacokinetics, Efficacy

## Abstract

Onivyde, a liposome-encapsulated irinotecan, is used for advanced pancreatic, while TAS-102 (trifluridine/tipiracil) is indicated for metastatic colorectal and gastric cancers. This study aims to determine the maximum tolerated dose (MTD), recommended phase II dose (RP2D), and safety profiles of liposomal irinotecan combined with TAS-102. This multicenter, phase I study utilized a 3 + 3 dose-escalation design. Patients with treatment-refractory solid malignancies received free base liposomal irinotecan at 50–70 mg/m^2^ on Day 1 and TAS-102 at 25–35 mg/m^2^ twice daily on Days 1–5 of a 14-day cycle. Patients homozygous for UGT1A1*28 (TA7/TA7), UGT1A1*6 (A/A), or double heterozygous (*TA6/TA7* and *G/A*) alleles were excluded. Prophylactic G-CSF was allowed. Twenty-six evaluable patients were enrolled across seven dose levels of liposomal irinotecan (free-base)/TAS-102 combination: 3 patients each at level 1 (50/25 mg/m^2^), level 2A (60/25 mg/m^2^), level 2B (50/30 mg/m^2^), and level 3 (60/30 mg/m^2^); 6 at level 4A (70/30 mg/m^2^); 3 at level 4B (60/35 mg/m^2^); and 5 at level 5 (70/35 mg/m^2^). An additional 15 patients were enrolled in the expansion cohort at the MTD of 70/30 mg/m^2^ (level 4A), designated as the RP2D. Overall grade 3–4 treatment-related adverse events occurred in 44.2% of 43 all treated patients, with neutropenia (16.3%), diarrhea (14%), and fatigue (11.6%). Partial responses were observed in 18.4% of patients, predominantly in neuroendocrine tumor, gastric and esophageal carcinomas. The combination of liposomal irinotecan and TAS-102 at the RP2D of 70/30 mg/m^2^ demonstrated acceptable safety and promising efficacy in refractory solid tumors, warranting further investigation.

## Background

Liposomal irinotecan (ONIVYDE, pegylated liposomal; formerly MM-398 or PEP02) uses a liposomal delivery system to encapsulate irinotecan, a topoisomerase I inhibitor that disrupts DNA unwinding, and inhibits cell proliferation [1, 2]. This formulation enhances pharmacokinetics and biodistribution, offering controlled release and extended circulation [3], which promotes drug accumulation in tumor tissues and improves therapeutic efficacy [4].

The Phase III NAPOLI-1 trial established the efficacy of liposomal irinotecan plus 5-FU/LV in gemcitabine-refractory metastatic pancreatic ductal adenocarcinoma (mPDAC), demonstrating a significant overall survival (OS) benefit over 5-FU/LV alone [5]. Building on this, NAPOLI-3 evaluated NALIRIFOX as a first-line regimen, showing superior OS and progression-free survival (PFS) compared to gemcitabine plus nab-paclitaxel in treatment-naïve mPDAC. Inspired by these results, ongoing trials are exploring oral alternatives like capecitabine or TS-1 to replace continuous 5-FU infusion [6, 7], leveraging their proven efficacy in colorectal cancer [8, 9]. This research highlights the potential of combining liposomal irinotecan with oral chemotherapy to enhance treatment accessibility and patient convenience.

TAS-102 is an orally available combination drug comprising trifluridine (FTD), a thymidine-based nucleoside analog, and tipiracil hydrochloride (TPI), a thymidine phosphorylase inhibitor that enhances FTD's anticancer effects [10]. The clinical utility of TAS-102 for treating refractory metastatic CRC was validated in the RECOURSE study, a multinational, randomized Phase 3 trial [11]. This study assessed TAS-102’s efficacy compared to best supportive care in CRC patients who had progressed despite all standard therapies, including 5-FU. The results demonstrated significant improvements in OS and PFS, which warranted further investigations into TAS-102's potential.

Liposomal irinotecan, commonly combined with 5-FU for PDAC treatment, has been explored with S-1 as an alternative [7, 12]. However, S-1’s tolerability issues in western populations and lack of US FDA approval limit its use [13]. To overcome these challenges, we are investigating liposomal irinotecan in combination with TAS-102 as a novel strategy to broaden treatment options for advanced solid tumors. A Phase I clinical trial has been initiated to determine the maximum tolerated dose (MTD) and recommended Phase II dose (RP2D), assess toxicity, efficacy, and pharmacokinetics, and explore the synergistic potential of this combination to improve treatment outcomes.

In the standard regimen for metastatic PDAC, liposomal irinotecan is given at 70 mg/m^2^ with 5-FU/LV (2400/400 mg/m^2^) every 2 weeks [14]. For refractory CRC, TAS-102 is approved at 35 mg/m^2^ twice daily (Days 1–5 and 8–12) every 4 weeks [11]. A prior phase I study in advanced CRC established the MTD of TAS-102 at 25 mg/m^2^ twice daily (Days 1–5 and 8–12) when combined with irinotecan 150 mg/m^2^ every 2 weeks [15]. Based on these findings, the current trial evaluates liposomal irinotecan (50–70 mg/m^2^) with TAS-102 (25–35 mg/m^2^ twice daily) to determine the optimal, tolerable combination for further clinical development.

## Method

### Study design

This Phase I, multicenter, open-label, single-arm study used a 3 + 3 dose-escalation design to determine the MTD and RP2D of liposomal irinotecan plus TAS-102. The MTD was defined as the highest dose level where ≤ 1 of 6 patients experienced a dose-limiting toxicity (DLT), assessed per CTCAE v5.0 during the first two treatment cycles. DLTs included Grade ≥4 neutropenia/lymphopenia lasting > 5 days despite G-CSF support, Grade ≥3 neutropenia with infection requiring intravenous antibiotics, severe thrombocytopenia requiring transfusion, Grade ≥4 anemia, Grade ≥3 non-hematologic toxicity (excluding anorexia, nausea, vomiting, and fatigue), as well as treatment delays > 2 weeks. The DLT observation period was 28 days.

**Fig. 1:**
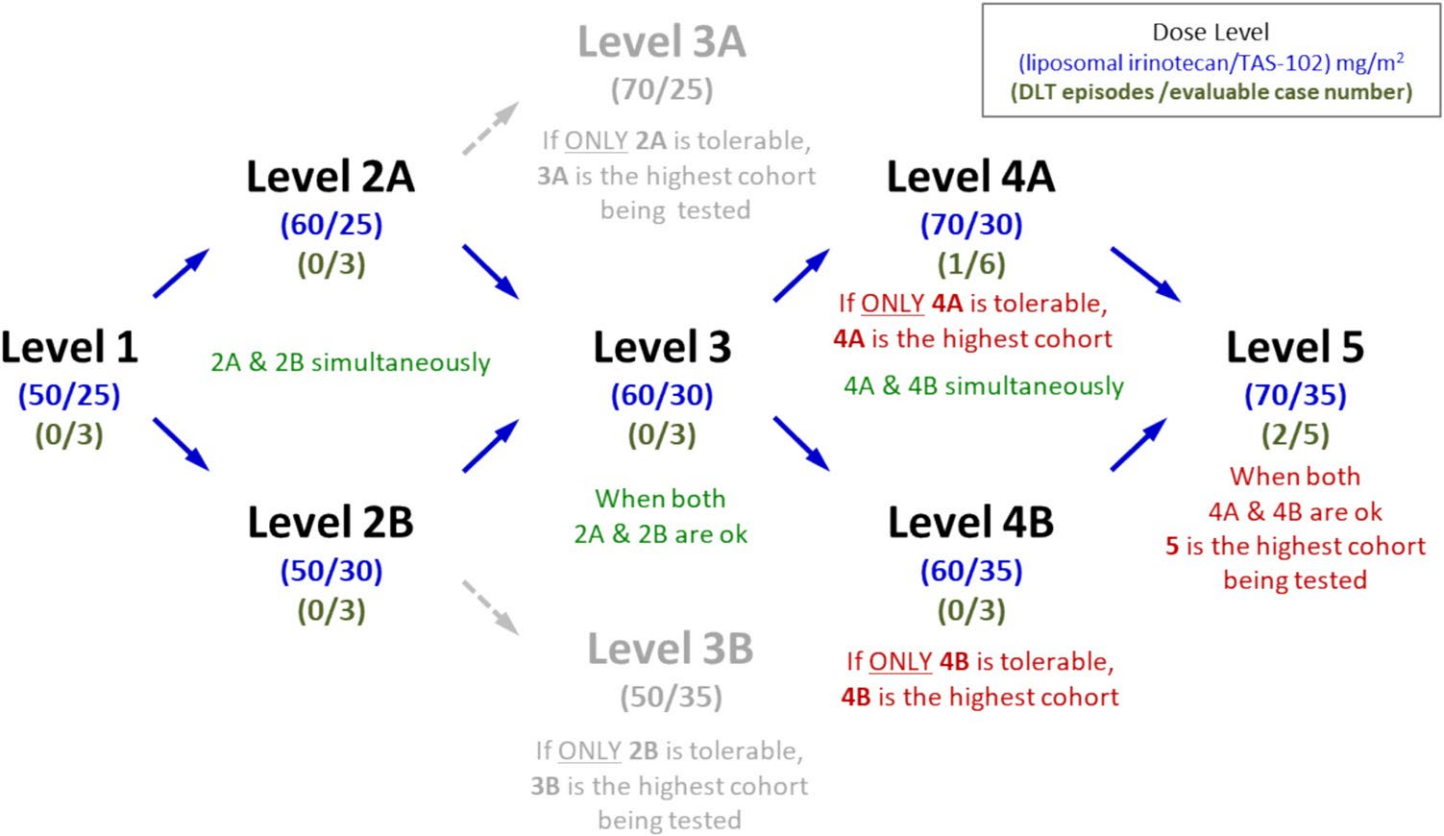
Scheme of Dose Escalation: Each level is labeled with the liposomal irinotecan (free-base)/TAS-102 doses (mg/m^2^) and the corresponding number of patients with dose-limiting toxicities (DLTs) versus patients treated in dose finding phase, shown in (DLT episodes/evaluable case number). For example, Level 1 (50/25) (0/3) indicates that 3 patients received liposamal irinotecan 50 mg/m^2^ plus TAS-102 25 mg/m^2^ without any DLT. Level 5 (70/35) (2/5) ultimately showed 2 DLTs among 5 patients, exceeding the threshold for declaring it the maximum tolerated dose

Patients received liposomal irinotecan (free base) infusions at 50–70 mg/m^2^ (equivalent to 60–80 mg/m^2^ as a salt-containing form) over 90 min on Day 1, with TAS-102 (25–35 mg/m^2^) taken orally twice daily on Days 1–5 of each 14-day cycle. Short-acting G-CSF was administered prophylactically for neutropenia. The study included five dose levels across nine cohorts (Fig. 1). After determining the MTD, an expansion cohort (10–15 patients) was enrolled at or below the MTD to confirm the RP2D. The study protocol was approved by each participating site's institutional review board (IRB), and all patients provided written informed consent.

### Study population

Eligible patients were adults (20–70 years) with advanced/metastatic solid tumors refractory to, or lacking, standard treatment. They required an ECOG performance status of 0–1 and adequate bone marrow (ANC ≥ 1500 cells/mm^3^, platelets ≥ 100,000 cells/mm^3^, hemoglobin ≥ 10 g/dL), hepatic, and renal function. Exclusion criteria included active CNS metastases, prior treatment with liposomal irinotecan or TAS-102, significant gastrointestinal disorders, and UGT1A1*28/*28* (TA7/TA7)*, UGT1A1*6/*6* (A/A)*, or* double heterozygous for both UGT1A1^*^28 allele (TA6/TA7) and UGT1A1^*^6 allele (G/A).

### Study aims

Primary objectives were to determine the MTD and the toxicity profile according to CTCAE v5.0. Secondary objectives included preliminary efficacy assessment (RECIST v1.1) and pharmacokinetics of the combination therapy.

### Pharmacokinetic sampling and analysis

In the expansion phase, a pharmacokinetic study was conducted during Cycle 1. Blood samples for both drugs were collected pre- and post-infusion on Days 1, 2, 5, and a single time point on Day 8. Pharmacokinetic parameters, including peak plasma concentration (C_max_), time to peak concentration (T_max_), half-life (T_1/2_), AUC from time zero to the time of the last quantifiable concentration (AUC_0-t_), AUC from time zero to infinity (AUC_0-∞_), and total clearance (CL), were analyzed using solid-phase extraction and liquid chromatography–mass spectrometry (Analyst v1.6.2, Watson v7.4.1).

### Safety analysis

All treated patients underwent safety assessments, including adverse events (AEs; graded per CTCAE v5.0), laboratory tests, vital signs, ECGs, ECOG performance status, and physical examinations. Treatment-emergent adverse events (TEAEs) were defined as AEs occurring or worsening after treatment initiation. A conservative algorithm classified events with incomplete onset dates as pre-treatment, treatment-emergent, or post-treatment. For recurring AEs, the highest CTCAE grade was recorded.

### Efficacy analysis

Efficacy was assessed per RECIST v1.1, with objective response rate (ORR) and disease control rate (DCR) as key endpoints. Patients without post-baseline tumor assessments were classified as non-responders. The duration of response (DoR) was summarized to describe response duration.

### Statistical method

Descriptive statistics were used. Continuous variables were summarized by mean, median, standard deviation (SD), and range, with changes from baseline presented with 95% confidence intervals (CIs). Categorical variables were reported by frequency and percentage, excluding missing data. Analyses were stratified by dose group and study phase. ORR and DCR were calculated for the all-treated and efficacy populations. Time-to-event outcomes were analyzed via Kaplan–Meier, with censoring at study end for patients without post-baseline assessments.

## Results

### Patient characteristics and dose escalation

Sixty-four patients were screened, with 44 (68.8%) enrolled and 43 treated. Patient allocation details are shown in Supplementary Figure S1. Of these, 29 participated in the dose-finding phase and 15 in the expansion phase, which included pharmacokinetic (PK) assessments.

In the dose-finding phase, patient distribution was as follows: Level 1 (n = 3), Level 2A (n = 3), Level 2B (n = 3), Level 3 (n = 3), Level 4A (n = 6), Level 4B (n = 4), and Level 5 (n = 6). One patient in Level 4B withdrew for personal reasons, and one in Level 5 discontinued due to an AE. DLTs were observed in one (grade 3 dehydration) of six patients at Level 4A (70 mg/m^2^ liposomal irinotecan and 30 mg/m^2^ TAS-102), none of the three evaluable patients at Level 4B (60 mg/m^2^ liposomal irinotecan and 35 mg/m^2^ TAS-102), dose escalation to Level 5 (70 mg/m^2^ liposomal irinotecan and 35 mg/m^2^ TAS-102) was performed. While DLTs were observed in two (grade 3 diarrhea and grade 4 sepsis one case each) of five evaluable patients at Level 5, no further enrollment was performed for Level 4B and Level 4A was selected for the expansion phase based on the decision of Data and Safety Monitoring Committee (DSMC), see the supplement Figure of dose escalation schema.

Patient demographics (Table 1) show a mean age of 53.7 years (range: 25–69), with most patients being male (62.8%) and of Asian descent. The most common cancer types were biliary tract (16.3%) and pancreatic malignancies. All patients had prior chemotherapy, averaging 3.4 cycles per patient.

**Table 1:** Baseline demographic and clinical characteristics

Characteristics	All Population N = 43
Number of patients	43
Age	
Mean ± SD	53.7 ± 10.8
Median (range), years	56 (25–69)
Sex	
Male	27 (62.8%)
Female	16 (37.2%)
ECOG PS	
0	30 (69.8%)
1	10 (30.2%)
BMI (kg/m2)	
Mean ± SD	23.8 ± 11.5
Median (range)	23 (17–32)
Cancer types	
Biliary tract	7 (16.3%)
Pancreas	4 (9.3%)
NET	3 (7.0%)
Ampulla of Vater	3 (7.0%)
Stomach	3 (7.0%)
Esophagus	3 (7.0%)
Sarcoma	3 (7.0%)
Thymic cancer	3 (7.0%)
Hepatocholangiocarcinoma	2 (4.7%)
Melanomas	2 (4.7%)
paraganglioma	2 (4.7%)
Breast	1 (2.3%)
Colorectum	1 (2.3%)
Endometrium	1 (2.3%)
Mesotheliomas	1 (2.3%)
Head & neck	1 (2.3%)
Ovary	1 (2.3%)
Small intestine	1 (2.3%)
Unknown primary	1 (2.3%)
Previous chemotherapies per patient	
Mean (SD)	3.4 (2.1)
Median (range)	3 (1–11)

N: number, SD: standard deviation, ECOG PS: eastern cooperative oncology group performance status, BMI: body mass index, NET/NEC: neuroendocrine tumor and carcinoma

### Safety

In the DLT-evaluable population, three patients experienced TEAEs meeting DLT criteria. One patient (16.7%) in Level 4 A (70 mg/m^2^ liposomal irinotecan and 30 mg/m^2^ TAS-102) had grade 3 dehydration, while two patients (40.0%) in Level 5 (70 mg/m^2^ liposomal irinotecan and 35 mg/m^2^ TAS-102) had grade 3 diarrhea and grade 4 sepsis, respectively. None of these were hematologic. Genetic analysis showed 15 (34.9%) all-treated patients had heterozygous UGT1A1*6, and 7 (16.3%) had UGT1A1*28 mutations. Among DLT cases, two (66.7%) had heterozygous *UGT1A1* mutations, one with UGT1A1*6 and one with UGT1A1*28.

All 43 patients experienced TEAEs (713 events), with 490 events in 41 patients (95.3%) related to study treatment. Most TEAEs (618 events, 42 [97.7%] patients) were grade 1–2, while 95 events in 25 (58.1%) patients were grade 3–5. Treatment-related adverse events (TRAEs) are summarized in Table 2, with 62 severe TRAEs affecting 19 (44.2%) patients. Common TRAEs included diarrhea (48.8%), nausea (44.2%), decreased WBC count (39.5%), vomiting (37.2%), fatigue (32.6%), neutropenia (30.2%), anemia (23.3%), and hypophagia (23.3%).

**Table 2:** Number (%) of Patients with Most Frequently Reported (≥ 10% of Patients) Treatment-Related AEs

Treatment-Related AEs	n (%) of all patients (N = 43)	
	All CTCAE Grades	CTCAE Grades 3–5
Any TRAEs	41 (95.3)	19 (44.2)
Hematologic toxicities		
White blood cell counts decreased	17 (39.5)	6 (14.0)
Neutrophil count decreased	13 (30.2)	7 (16.3)
Platelet count decreased	6 (14.0)	-
Anemia	10 (23.3)	1 (2.3)
Non-hematologic toxicities		
Gastrointestinal disorders	34 (79.1)	6 (14.0)
Diarrhea	21 (48.8)	6 (14.0)
Nausea	19 (44.2)	-
Vomiting	16 (37.2)	-
Abdominal pain	6 (14.0)	-
General disorders and administration site conditions	22 (51.2)	6 (14.0)
Fatigue	14 (32.6)	5 (11.6)
Metabolism and nutrition disorders	16 (37.2)	2 (4.7)
Hypophagia	10 (23.3)	2 (4.7)
Skin and subcutaneous tissue disorders	6 (14.0)	-

TRAEs: Treatment-Related Adverse Events; CTCAE: Common Terminology Criteria for Adverse Events; AEs: Adverse Events

Two patient deaths (due to pneumonia and sudden death) occurred but were not attributed to the study drug. TEAEs led to discontinuation in three patients (7.0%), including gastrointestinal hemorrhage, ileus, neutropenia, and pneumonia. Treatment modifications were required in 28 (65.1%) patients, with 5 (11.6%) experiencing interruptions and 25 (58.1%) requiring dose reductions or delays. Grade 3–5 TEAEs necessitated dose adjustments for liposomal irinotecan in 20 (46.5%) patients and TAS-102 in 17 (39.5%) patients. No unexpected safety signals emerged.

The primary reason for treatment discontinuation was disease progression (58.1%), affecting 21 patients per RECIST 1.1 and 4 with clinical progression. Additionally, 13 (30.2%) withdrew consent, 2 (4.7%) died, and 3 (6.9%) discontinued due to AEs or physician decisions.

### Pharmacokinetics

Pharmacokinetic parameters of total irinotecan, its active metabolite SN-38, trifluridine (FTD), and tipiracil hydrochloride (TPI) were assessed in an expansion cohort of 15 patients with refractory solid tumors. These patients received a single dose of liposomal irinotecan (70 mg/m^2^) administered as a 1.5-h intravenous infusion on Day 1 of Cycle 1, coupled with a twice-daily oral dose of TAS-102 (30 mg/m^2^) from Day 1 to Day 5 of the same cycle. Detailed Pharmacokinetic parameters are summarized in Table 3.

**Table 3:** Plasma Pharmacokinetic Parameters for single 1.5-Hour Infusion of 70 mg/m^2^ liposomal Irinotecan and twice Daily oral Administration of 30 mg/m^2^ TAS-102 in Patients with Refractory Solid Tumors (PK Population)

	C_max_	T_max_ (hr)	AUC_0-t_	AUC_0–8_	AUC_0-τ_	AUC_0-∞_	T_1/2_ (hr)	λz (1/hr)	CL (L/h/m^2^)	Vd (L/m^2^)	AR
CPT-11	31 ± 7.16 (μg/mL)	2.06 ± 0.84	604 ± 394 (h*μg/mL)	-	-	617 ± 398 (h*μg/mL)	22.3 ± 12.8	0.0478 ± 0.037	0.207 ± 0.152	4.87 ± 2.81	-
SN-38	4.29 ± 1.76 (ng/mL)	21.14 ± 22.2	328 ± 209 (h*ng/mL)	-	-	396 ± 238^a^ (h*ng/mL)	48.2 ± 28.7^a^	0.0193 ± 0.01^a^	-	-	-
FTD (Day 1)	1900 ± 828^b^ (ng/mL)	1.7 ± 1.11^b^	4430 ± 1440^b^ (h*ng/mL)	5020 ± 1160 (h*ng/mL)	-	5200 ± 1260^b,c^ (h*ng/mL)	1.36 ± 0.480^c^	0.566 ± 0.177^b,c^	CL/F (L/h/m^2^)	Vd/F(L/m^2^)	-
									6.15 ± 1.78 ^b,c^	11.4 ± 3.28^b,c^	
FTD (Day 5)	3200 ± 1430^d^ (ng/mL)	2.26 ± 1.49^d^	11600 ± 3150^d^ (h*ng/mL)	-	11900 ± 3440^d,e^ (h*ng/mL)	-	2.14 ± 0.943^d,f^	0.364 ± 0.124^d,f^	CLss/F (L/h/m^2^)	Vss/F (L/m^2^)	3.34 ± 2.04
									2.75 ± 0.945^d,e^	6.91 ± 2.93^d,f^	
TPI (Day 1)	54.5 ± 26.0^b^(ng/mL)	3.2 ± 1.53^b^	220 ± 117^b^ (h*ng/mL)	386 ± 1.67^b^ (h*ng/mL)		486 ± 71.4^b,g^ (h*ng/mL)	3.05 ± 1.28^b,g^	0.249 ± 0.104^b,g^	CL/F (L/h/m^2^)	Vd/F (L/m^2^)	-
									25.6 ± 3.76^b,g^	109 ± 30.6^b,g^	
TPI (Day 5)	51.6 ± 25.4^d^ (ng/mL)	3.22 ± 1.30^d^	191 ± 108 ^d^ (h*ng/mL)	-	165 ± 130 ^d,h^ (h*ng/mL)	-	1.60 ± 0.0672 ^d,i^	0.434 ± 0.018^d,i^	CLss/F (L/h/m^2^)	Vss/F (L/m^2^)	0.984 ± 0.441
									117 ± 79.9 ^d,h^	200 ± 78.7 ^d,i^	

Mean ± STD; CPT-11: Irinotecan, FTD: Trifluridine, TPI: Tipiracil hydrochloride, C_max_: Maximum serum concentration, T_max_: Time when maximum serum concentration was observed, AUC_0-t_: Area under the concentration–time curve from time zero to time of last quantifiable concentration, AUC_0-τ_: Area under the concentration–time curve from time zero to tau at steady state, where tau is the dosing interval, AUC_0-∞_: Area under the concentration–time curve from time zero to infinity, T_1/2_: Half-life, λz: Terminal elimination rate constant, CL: Total clearance, CL/F: Total apparent clearance, CLss/F: Total apparent clearance at steady state, Vd: Volume of distribution based on terminal phase, Vd/F: Apparent volume of distribution based on terminal phase, Vss/F: Apparent volume of distribution based on terminal phase at steady state, AR: accumulation ratio, calculated as AUC_0-τ_ (Day 5)/AUC_0–8_ (Day 1); whenever either AUC_0-τ_ or AUC_0–8_ was not estimable, AUC_0-t_ was used alternatively

a: In SN-38, AUC_0-∞_, T_1/2_, and λz could not be estimated for one subject due to the positive slope in the terminal phase

b: In FTD (Day 1) and TPI (Day 1), one subject was excluded due to the protocol violation, TAS-102 was not taken at about the time when the infusion of liposomal irinotecan started. Another subject was excluded due to vomiting at or before 2 times median T_max_

c: In FTD (Day 1), AUC_0-∞_, T_1/2_, λz, CL/F, and Vd/F could not be estimated for 4 subjects due to less than three measurable concentrations after C_max_

d: In FTD (Day 5) and TPI (Day 5), one subject was excluded due to the incomplete PK profile; three subjects were excluded due to vomiting at or before 2 times median T_max_

e: In FTD (Day 5), AUC_0-τ_ and CLss/F could not be estimated for 3 subjects because the sampling ended earlier than the end of doing interval

f: In FTD (Day 5), T_1/2_, λz, and V_ss_/F could not be estimated for 7 subjects due to less than three measurable concentrations after C_max_

g: In TPI (Day 1), AUC_0-∞_, T_1/2_, λz, CL/F, and Vd/F could not be estimated for 11 subjects due to less than three measurable concentrations after C_max_

h: In TPI (Day 5), AUC_0-τ_ and CLss/F for 4 subjects could not be estimated because the sampling ended earlier than the end of doing interval

i: In TPI (Day 5), T_1/2_, λz, and Vss/F could not be estimated for 9 subjects due to less than three measurable concentrations after C_max_

Following a 1.5-h intravenous infusion of liposomal irinotecan, peak plasma concentrations of total irinotecan were observed at the completion of the infusion, with average C_max_ and T_max_ values of 31.0 μg/mL and 2.06 h, respectively. Plasma irinotecan declined gradually, with a half-life of approximately 22.3 h. The overall exposure to irinotecan quantified as the mean area under the curve from time zero to infinity (AUC_0-∞_) was 617 h*μg/mL, with mean clearance and volume of distribution values reported at 0.207 L/h/m^2^ and 4.87 L/m^2^, respectively. SN-38, the active metabolite of irinotecan, reached a maximum plasma concentration of approximately 4.29 ng/mL, with a mean T_max_ occurring 21.14 h post-infusion and a half-life of about 48.2 h. The mean AUC_0-∞_ for SN-38 was calculated to be 396 h*ng/mL.

Regarding TAS-102, a noticeable accumulation of FTD was observed after twice daily oral administration of 30 mg/m^2^ for five days, with a mean accumulation ratio of 3.34, indicating a significant increase in concentration over time. The maximum plasma concentration increased from 1900 ng/mL on the first day (T_max_ 1.70 h), to 3200 ng/mL on Day 5 (T_max_: 2.26 h). The half-life also extended from approximately 1.36 h to about 2.14 h. Conversely, TPI showed no accumulation with a mean accumulation ratio of 0.98, maintaining a relatively stable peak plasma concentration of about 54.5 ng/mL on the first day (T_max_: 3.20 h) and slightly decreasing to around 51.6 ng/mL on Day 5 (T_max_: 3.22 h), with a half-life decreased from approximately 3.05 h to 1.60 h.

### Antitumor activity

**Fig. 2:**
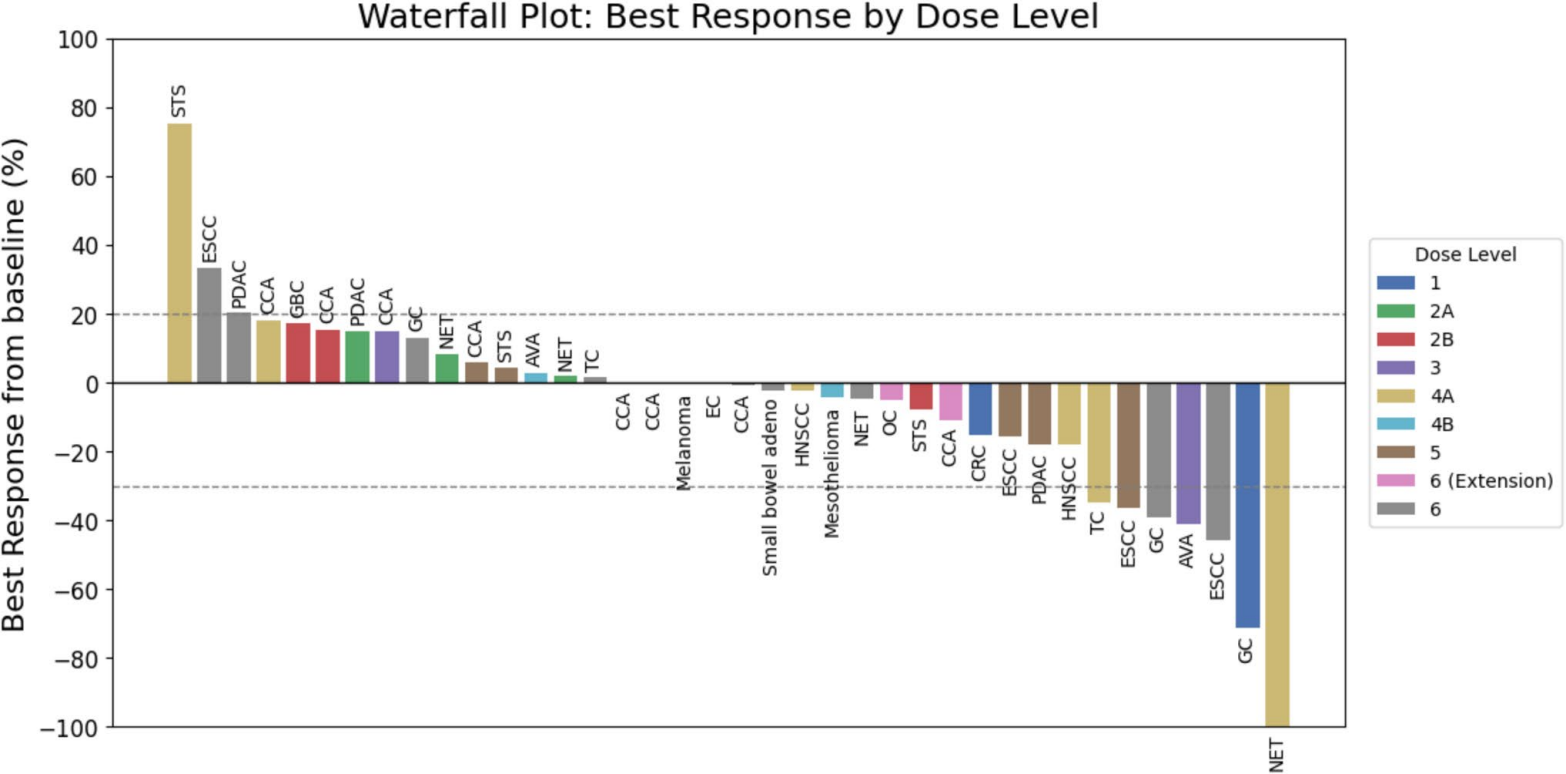
Waterfall Plot—Percentage of Change in the Tumor Size from Baseline to Best Assessment Based on the Best Target Lesion Response (Efficacy Population, N = 38) AVA, ampulla Vater adenocarcinoma; CCA, cholangiocarcinoma; CRC, colorectal cancer; EC, endometrial carcinoma; ESCC, esophageal squamous cell carcinoma; GBC, gallbladder cancer; GC, gastric cancer; HNSCC, head and neck squamous cell carcinoma (tonsillar cancer); Melanoma, malignant melanoma; Mesothelioma, malignant mesothelioma; NET, neuroendocrine tumor; OC, ovarian cancer; PDAC, pancreatic ductal adenocarcinoma; Small bowel adeno, small bowel adenocarcinoma; STS, soft tissue sarcoma; TC, thymic carcinoma

The treatment efficacy outcomes of the 38 patients who underwent at least one post-treatment tumor assessment were included in the efficacy population and the details are shown in Table 3S. Among the 38 patients, no complete response (CR) was observed, 7 (18.4%) patients achieved a partial response (PR), 25 (65.8%) patients had stable disease (SD), and 6 (15.8%) patients exhibited progressive disease (PD). The overall ORR was 18.4%, and the DCR reached 84.2%. One patient with treatment-refractory grade 3 neuroendocrine tumor (NET) of the rectum, characterized by a Ki-67 index of 30%, achieved PR after 4 months, and then nearly CR after 25 months of treatment. The median progression-free survival (PFS) for both efficacy and all-treated populations stood at 30.0 weeks, with a 95% CI of 12.0–48.3 weeks. The mean and median DoR across the cohorts was 29.8 and 18 weeks, respectively. Figure 2S shows a detailed swimmer plot, illustrating the best overall response and progression of the disease by patients. Figure 2 presents a waterfall plot that depicts the percentage change in tumor size from baseline to best assessment in the efficacy population.

## Discussion

This phase I study is the first to evaluate the safety, tolerability, and preliminary efficacy of liposomal irinotecan combined with TAS-102 in patients with advanced solid tumors. With prophylactic use of G-CSF, both Level 4A and Level 4B were tolerable, with DLT being observed in one of six and none of three patients, respectively. Level 4A (70 mg/m^2^ of liposomal irinotecan plus 30 mg/m^2^ of TAS-102, twice daily, Days 1–5, in a 14-day treatment cycle) was selected as RP2D based on the decision of DSMC. TRAEs were observed in 95.3% of participants, with the majority (86.7%) being grade 1–2 per CTCAE criteria. The most reported TRAEs were gastrointestinal events, including diarrhea, nausea, vomiting, and abdominal discomfort, aligning with the known safety profiles of both agents [5, 11, 16, 17].

In the current study, although the RP2D of the combination was selected to be 70 mg/m^2^ liposomal irinotecan combined with 30 mg/m^2^ TAS-102 (Level 4A), the absence of DLT in the three patients at Level 4B supports that 60 mg/m^2^ liposomal irinotecan plus 35 mg/m^2^ TAS-102 could be served as an alternative dosing regimen, consist with that reported in the OniLon trial [18].

In this study, prophylactic short-acting G-CSF was incorporated based on the anticipated hematologic toxicity of both TAS-102 and liposomal irinotecan, particularly in Asian populations known to exhibit higher susceptibility to myelosuppression. For instance, in the pivotal trial of TAS-102 for metastatic gastric cancer, grade 3–4 neutropenia was reported in 52.2% of Japanese patients [19]. Similarly, in the NAPOLI-1 trial, liposomal irinotecan at 70 mg/m^2^ combined with 5-FU/LV was associated with a markedly higher incidence of Grade 3–4 neutropenia in the Asian subgroup (54.5%) compared to the overall study population (27.4%) [20]. In a Japanese Phase I study evaluating TAS-102 plus conventional irinotecan, the recommended dose was 25 mg/m^2^ twice daily for TAS-102 and 150 mg/m^2^ for irinotecan, with grade 3–4 neutropenia and febrile neutropenia occurring in 100% and 30% of patients, respectively [15]. Given the overlapping myelotoxicity of TAS-102 and liposomal irinotecan, prophylactic G-CSF was used to ensure safe dose escalation and maintain dose intensity. This likely contributed to the lower incidence of grade 3–4 neutropenia compared to other Asian studies without routine G-CSF. While this adds clinical and economic burden, its use was justified in a phase I setting prioritizing safety. Further studies are warranted to assess tolerability without G-CSF in real-world settings.

Previous studies have established key benchmarks for the use of liposomal irinotecan in various treatment settings. The PEP0201 study identified the MTD of liposomal irinotecan monotherapy as 100 mg/m^2^ (equivalent to 120 mg/m^2^ as the salt-containing form) every three weeks, demonstrating a manageable toxicity profile and potential for combination therapy [17]. Additionally, the PEP0203 phase I study, which determined the MTD of liposomal irinotecan as 70 mg/m^2^ at day 1 in combination with 24-h infusion of 5-FU/LV at Days 1 and 8 on a three-week cycle in solid tumors. Of the 6 patients at MTD dose level, only 10.6% experienced grade ≥ 3 AEs [21]. The lower incidence of severe AEs observed in PEP0203 compared to the present study may be attributed to the longer treatment intervals. In contrast, the NIFTY study, which evaluated liposomal irinotecan (70 mg/m^2^) plus 5-FU/LV every two weeks in metastatic cholangiocarcinoma, reported Grade ≥ 3 adverse events in 42% of patients, with neutropenia being the most common [22]. In our study, the overall AE rates were comparable. However, grade 3 diarrhea occurred more frequently (14%) with TAS-102 in our study compared to 5% in NIFTY and 3% of Asian subpopulation in NAPOLI-1 suggesting that TAS-102 may contribute to an increased gastrointestinal toxicity burden compared to 5-FU/LV [20, 22]. This highlights the importance of proactive gastrointestinal management in future studies evaluating this combination.

Pharmacokinetic analysis revealed that when TAS-102 was administered with 70 mg/m^2^ liposomal irinotecan, the C_max_ of FTD on Day 1 was 1900 ± 828 ng/mL, which is approximately half of the reported C_max_ (3677 ± 1459 ng/mL) observed in prior TAS-102 monotherapy studies at the same dose [23]. Similar reductions were observed for TPI, with a C_max_ of 54.5 ± 26.0 ng/mL in the combination therapy, compared to 136 ± 77 ng/mL with TAS-102 monotherapy. These findings suggest a potential pharmacokinetic interaction between liposomal irinotecan and TAS-102, possibly affecting the bioavailability of FTD and TPI. Further supporting this, a Phase I study evaluating TAS-102 (25 mg/m^2^ BID) in combination with irinotecan and bevacizumab reported a Day 1 FTD C_max_ of 3,362 ± 1,702 ng/mL, significantly higher than the levels observed in our study [24]. The variations in pharmacokinetic parameters across different studies suggest that individual patient factors, drug combinations, and dosing schedules may significantly impact FTD and TPI exposure.

The PEP0203 study reported CPT-11 C_max_ of 29.2 ± 5.2 µg/mL and SN-38 C_max_ of 7.98 ± 4.39 ng/mL at 70 mg/m^2^ liposomal irinotecan with 5-FU/LV [21]. In our study, at the same dose level, CPT-11 C_max_ (31 ± 7.16 µg/mL) was comparable, while SN-38 levels (4.29 ± 1.76 ng/mL) were somewhat lower but still within a similar range when considering standard deviations. These variations could be attributed to differences in patient populations, sampling time points, or subtle protocol discrepancies between studies. Despite these differences, both data sets confirm that a 70 mg/m^2^ dose of liposomal irinotecan provides SN-38 exposure in a comparable range, supporting the feasibility of this dosing strategy in combination regimens. Furthermore, our PK analysis demonstrated that SN-38 exhibited a prolonged half-life (~ 48.2 h), consistent with the expected sustained circulation of liposomal irinotecan, compared to conventional irinotecan formulations (~ 12–22 h) [25]. However, considerable inter-patient variability in SN-38 clearance was observed, which may contribute to differences in treatment-related toxicities across patients.

Efficacy analysis of our study demonstrated a partial response (PR) rate of 18.4% and a DCR of 84.2%, with a median DoR of 18 weeks and median PFS of 30 weeks. These results compare favorably with previous studies evaluating irinotecan-based regimens. In the PEP0203 study, which examined liposomal irinotecan plus 5-FU/LV every three weeks, 13.3% of patients achieved PR, and the DCR was 73.3% [21]. Similarly, the TABASCO phase II trial, investigating TAS-102 with irinotecan and bevacizumab, reported an ORR of 18.4% and a median PFS of 7.9 months, albeit with a higher incidence of grade ≥ 3 toxicities (67%), primarily neutropenia and gastrointestinal events [26]. Another phase I study in Japanese patients identified a recommended dose of 50 mg/m^2^/day TAS-102 plus 150 mg/m^2^ irinotecan, reporting a partial response rate of 22% and neutropenia as the most frequent grade 3–4 toxicity [15]. The OniLon phase II trial, which evaluated liposomal irinotecan plus TAS-102 in metastatic colorectal cancer, reported an ORR of 15%, a DCR of 75%, and a median PFS of 9.7 months, further reinforcing the clinical potential of TAS-102 combined with irinotecan-based regimens [27].

The favorable antitumor activity observed in our study may be attributed to shorter treatment intervals and the addition of TAS-102, which has shown efficacy even in 5-FU-pretreated patients [11, 28]. Notably, two of the seven PR cases were in gastric cancer patients, suggesting potential clinical relevance in this setting. In current clinical practice, taxane-based therapy is the standard second-line treatment after platinum-based chemotherapy failure, with irinotecan as an alternative for patients with taxane intolerance due to neuropathy [29]. The TAGS trial has already validated TAS-102 as a third-line option for gastric cancer, demonstrating improvements in OS, PFS, and quality of life [28]. These findings support further evaluation of liposomal irinotecan plus TAS-102 as a second-line or later-line therapy for gastric cancer, forming the basis for an ongoing phase II study (NCT05927857) investigating this combination with ramucirumab.

## Conclusion

This Phase I trial represents one of the earliest investigations of liposomal irinotecan (Onivyde) combined with TAS-102 in advanced solid tumors, establishing a manageable safety profile and promising antitumor activity. With prophylactic use of G-CSF, 70 mg/m^2^ of liposomal irinotecan plus orally 30 mg/m^2^ of TAS-102, twice daily, Days 1–5, in a 14-day treatment cycle was selected as RP2D based. These findings provide a strong foundation for further clinical development, supporting further trials aimed at expanding therapeutic options for gastrointestinal malignancies.

## Data Availability

No datasets were generated or analysed during the current study.
